# MOCART 2.0 score of 60 or greater measured at 1 year post‐operatively predicts favourable clinical outcomes after surgical repair of tibiofemoral cartilage lesions

**DOI:** 10.1002/ksa.70086

**Published:** 2025-10-15

**Authors:** Hyun‐Soo Moon, Sungjun Kim, Chong‐Hyuk Choi, Min Jung, Kwangho Chung, Se‐Han Jung, Jin‐Gyu Kim, Min‐Cheol Park, Sung‐Hwan Kim

**Affiliations:** ^1^ Arthroscopy and Joint Research Institute Yonsei University College of Medicine Seoul Republic of Korea; ^2^ Department of Orthopedic Surgery, Gangnam Severance Hospital Yonsei University College of Medicine Seoul Republic of Korea; ^3^ Department of Radiology, Gangnam Severance Hospital Yonsei University College of Medicine Seoul Republic of Korea; ^4^ Department of Orthopedic Surgery Yonsei Bonsarang Hospital Bucheon Republic of Korea; ^5^ Department of Orthopedic Surgery, Severance Hospital Yonsei University College of Medicine Seoul Republic of Korea; ^6^ Department of Orthopedic Surgery, Yongin Severance Hospital Yonsei University College of Medicine Yongin Republic of Korea

**Keywords:** cartilage repair, clinically important difference, cut‐off value, MOCART 2.0 score, substantial clinical benefit, tibiofemoral joint

## Abstract

**Purpose:**

(1) To evaluate the relationship between the Magnetic Resonance Observation of Cartilage Repair Tissue (MOCART) 2.0 score and post‐operative clinical outcomes following surgical repair of cartilage lesions in the tibiofemoral joint, and (2) to determine threshold values of the 1‐year MOCART 2.0 score associated with favourable patient‐reported outcome measures (PROMs).

**Methods:**

Medical records of patients who underwent surgical repair for tibiofemoral joint cartilage lesions from 2010 to 2022 were retrospectively reviewed, and those who had magnetic resonance imaging and clinical assessments 1 year post‐operatively were included. Outcomes were assessed using the International Knee Documentation Committee subjective score, Lysholm score and Knee Injury and Osteoarthritis Outcome Score (KOOS), with respective clinically important difference (CID) and substantial clinical benefit (SCB) values used to evaluate clinically significant improvement. Relationships between MOCART 2.0 scores and PROMs were analyzed, with cut‐off values determined via receiver operating characteristic (ROC) analysis, followed by group comparisons.

**Results:**

Eighty‐six patients were included (mean age, 51.9 ± 14.3 years; males/females, 16/70; mean lesion size, 3.1 ± 1.5 cm^2^). The MOCART 2.0 score showed positive correlations with most PROMs at 1 year post‐operatively. Logistic regression revealed significant associations between MOCART 2.0 scores and clinical improvements beyond CID and SCB values for the Lysholm score and SCB values for KOOS symptoms, which showed similar trends even when threshold values were adjusted by ±5 points in analyses. Subsequent ROC curve analyses identified statistically significant cut‐off points ranging from 56 to 61 points. Comparative analysis, classified using a threshold of 60 considering its scoring system, demonstrated that patients with scores ≥60 showed generally higher PROMs and lower osteoarthritis grades at 1 year post‐operatively and final follow‐ups.

**Conclusions:**

The MOCART 2.0 score, assessed 1 year after surgical repair for cartilage lesions in the tibiofemoral joint, positively correlates with PROMs, with scores of ≥60 expected to be associated with favourable clinical outcomes.

**Level of Evidence:**

Level IV.

**Abbreviations**
ACI: autologous chondrocyte implantation
CID: clinically important difference
FS: fat‐saturated
ICC: intraclass correlation coefficient
IKDC: International Knee Documentation Committee
KOOS: Knee Injury and Osteoarthritis Outcome Score
MOCART: Magnetic Resonance Observation of Cartilage Repair Tissue
MRI: magnetic resonance imaging
MSC: mesenchymal stem cell
MSP: marrow stimulation procedure
PD: proton density
PROM: patient‐reported outcome measure
ROC: receiver operating characteristic
SCB: substantial clinical benefit
TSE: turbo spin‐echo

## INTRODUCTION

With the increasing frequency of surgical repair for cartilage lesions in the knee joint, the importance of radiological evaluation to assess surgically repaired cartilage has become more prominent [5, 6, 8, 20]. Previously, the Magnetic Resonance Observation of Cartilage Repair Tissue (MOCART) and three‐dimensional MOCART scoring system were widely used for the morphological assessment of repair tissue, providing objective and semiquantitative analysis [7, 12, 16, 26]. However, advances in surgical techniques and magnetic resonance imaging (MRI) necessitated an improved tool, leading to the recent introduction of the MOCART 2.0 score, which demonstrates high measurement reliability and addresses limitations of earlier systems [19, 24, 25].

Nevertheless, despite the potential benefits of the MOCART 2.0 scoring system, its clinical relevance and the interpretative guidelines correlated with clinical outcomes have yet to be fully established. While it is logical to expect that favourable morphological assessments of repair tissue would correlate with positive clinical outcomes [2], there is currently little scientific evidence to validate this expectation for this scoring system. Furthermore, the criteria for clinically interpreting the MOCART 2.0 score, particularly with respect to its correlation with clinical outcomes, are still underdeveloped. It remains unclear the specific score threshold needed to reliably indicate successful repair or predict favourable clinical outcomes. Without the aforementioned information, the clinical significance of using this scoring system remains limited.

Therefore, this study aimed to (1) evaluate the relationship between the MOCART 2.0 score and post‐operative clinical outcomes following surgical repair for cartilage lesions in the tibiofemoral joint, and (2) determine relevant threshold values correlated with these clinical outcomes. It was hypothesized that the MOCART 2.0 score would show a positive correlation with patient‐reported outcome measures (PROMs) and that there would be a specific cut‐off value predicting favourable outcomes.

## METHODS

Approval for this study was granted by the institutional review board of our institution, with the need for informed consent being waived due to the study's retrospective design (ID Number: 4‐2024‐0996). The medical records of patients who underwent surgical repair for cartilage lesions of the knee joint by a senior surgeon at a single institution between 2010 and 2022 were retrospectively reviewed. To be eligible for inclusion in this study, patients had to have undergone surgical repair for cartilage lesions in the tibiofemoral joint, followed by MRI assessment one year post‐operatively, with clinical scores evaluated at the same time point. Patients meeting the following criteria were excluded from the study: (1) absence of follow‐up MRI or MRI of insufficient quality to evaluate the MOCART 2.0 score (e.g., poor‐quality imaging or missing specific sequences) [24], (2) loss to follow‐up after surgery, (3) reoperation, (4) surgical repair for cartilage lesions of the patellofemoral joint, (5) post‐operative infection and (6) concomitant surgeries, including osteotomy, ligament, and meniscus surgery. These concomitant procedures were excluded to avoid their potential influence on clinical outcomes independent of cartilage repair. Additionally, patients with cartilage lesions smaller than 1.5 cm^2^ at the time of surgery were excluded, as smaller lesions may impact the accuracy of MRI‐based assessments.

### Types of surgical procedures and rehabilitation methods

In our institution, cartilage repair was generally considered for relatively young and active patients when MRI revealed that the depth of the cartilage lesion extended to the calcified layer, accompanied by relevant clinical symptoms. The recommended treatment options included marrow stimulation procedures (MSPs), enhanced MSP, autologous chondrocyte implantation (ACI) and mesenchymal stem cell (MSC) implantation. MSP involved either microfracture or microdrilling techniques, while enhanced MSP utilized biomaterial scaffolds in addition to MSP [12, 19]. ACI referred to the third‐generation procedure (CartiLife®, Biosolutions Co., Ltd.), and MSC implantation used allogeneic cells derived from umbilical cord tissue (Cartistem®, MediPost Co., Ltd.) [10, 27]. During preoperative consultation, patients were informed about the characteristics, indications, prognosis and costs of each surgical technique, allowing them to make an informed decision regarding their treatment.

Post‐operatively, regardless of the specific cartilage repair technique used, joint protection measures were implemented for 8 weeks using crutches and a hinged knee brace. For gait, patients wore the hinged knee brace in full knee extension while ambulating with crutches. Toe‐touch weight‐bearing was allowed for the first 4 weeks, followed by partial weight‐bearing (less than 50% of body weight) for the next 4 weeks. After this period, full weight‐bearing without the brace and crutches was permitted. Passive range of motion exercises were encouraged immediately after surgery, while active range of motion exercises began 8 weeks post‐operatively. Strengthening exercises were introduced from the 8th week, once full weight‐bearing and active range of motion exercises were allowed, starting with closed kinetic chain exercises, with open kinetic chain exercises being permitted later in the rehabilitation process. Jogging, running, and other dynamic activities, including sports participation, were generally permitted from 6 months post‐operatively.

### Patient assessments

The MOCART 2.0 score was assessed using MRI performed one year after surgery as part of routine clinical follow‐up, except for patients who declined the examination. All MRIs were conducted on a 3.0 Tesla system (General Electric and Achieva, Philips Healthcare), and a coronal fat‐saturated (FS) proton density (PD) weighted turbo spin‐echo (TSE) sequence and a sagittal non‐FS PD‐weighted TSE sequence were utilized for the morphological assessment of the surgical repair site [24]. The seven variables constituting the MOCART 2.0 score were measured, and the total score was used for analysis. In addition to the MRI‐based evaluation, lower limb alignment and osteoarthritis grade were assessed using plain radiographs. Preoperative lower limb alignment was evaluated by measuring the hip–knee–ankle angle on full‐length, weight‐bearing anteroposterior radiographs of both lower extremities [15]. For the osteoarthritis grade, standing anteroposterior knee radiographs obtained at the preoperative, 1 year post‐operative and final follow‐up time points were graded according to the Kellgren–Lawrence system [11]. Radiological evaluations were independently performed on a picture archiving and communication system workstation by two experienced orthopaedic surgeons, each blinded to all patient information and to the other observer's measurements. For continuous variables, the average of the two measurements was used in the analysis. For categorical variables, any discrepancies between observers were discussed until consensus was reached; in cases where no agreement could be achieved, a decision was made in consultation with the senior author.

The PROMs used for evaluation in this study included the International Knee Documentation Committee (IKDC) subjective score, the Lysholm score, and the Knee Injury and Osteoarthritis Outcome Score (KOOS) [1, 9, 13]. These were obtained from medical records at the preoperative visit, the 1‐year post‐operative follow‐up, and as well as the final follow‐up. To determine whether clinically significant improvement was achieved at 1 year post‐operatively compared to preoperative status, established respective clinically important difference (CID) values and substantial clinical benefit (SCB) values were used as evaluation thresholds for each clinical score (Table 1) [3, 21].

**Table 1:** CID and SCB of the IKDC subjective score, Lysholm score and KOOS.

Variables	CID^a^	SCB^b^
IKDC subjective score	9.2	34.4
Lysholm score	13.0	29.0
KOOS symptoms	10.7	14.3
KOOS pain	8.3	27.7
KOOS activities of daily living	8.8	29.4
KOOS sports and recreation	30.0	30.0
KOOS quality of life	18.8	37.5

Abbreviations: CID, clinically important difference; IKDC, International Knee Documentation Committee; KOOS, Knee injury and Osteoarthritis Outcome Score; SCB, substantial clinical benefit.

^a^ Values references from the study by Chahal et al.

^b^ Values references from the study by Ogura et al.

Additionally, the intraoperative data were analyzed based on operative records, including the affected compartment of the tibiofemoral joint (the location of the cartilage lesion), cartilage lesion size, type of cartilage repair surgery, and the condition of the medial and lateral menisci. The affected compartment was classified as either medial or lateral. Cartilage lesion size was measured intraoperatively using an arthroscopic hook probe (Arthrex, Hook 5.4 mm, Tip with 5‐mm markings; AR‐10000), referencing the 5‐mm interval markings on the tip and shaft. Meniscal status was recorded separately for the medial and lateral meniscus and categorized as functional, non‐functional or repaired. A meniscus was considered functional when no pathological lesion was present or when partial meniscectomy had been performed for a condition that did not impair its function [18]. A non‐functional meniscus was defined as one with a history of prior meniscectomy or with extensive irreparable lesions [18].

### Statistical analysis

Prior to conducting the analyses, an a priori sample size calculation was performed. Considering the primary objective of this study, a power analysis was conducted for the correlation analysis, with a power of 0.8 and a correlation coefficient (*r*) of 0.3. The results indicated that a minimum sample size of 85 participants was required to adequately assess the relationship between the MOCART 2.0 score and post‐operative clinical outcomes following surgical repair of cartilage lesions in the tibiofemoral joint.

All statistical analyses were performed using IBM SPSS Statistics for Windows (v26.0). Pearson correlation analysis was employed to examine the relationship between the MOCART 2.0 score and PROMs at 1 year post‐operatively. Logistic regression was conducted to evaluate whether the MOCART 2.0 score influenced the achievement of clinically significant improvement, as defined by exceeding the respective CID and SCB values for each PROM at one year post‐operatively compared to preoperative status [3, 21]. Moreover, to address the uncertainty surrounding the use of reported CID and SCB threshold values for group classification, this study conducted additional exploratory analyses with adjusted thresholds. Specifically, new regression models were developed by modifying the original CID and SCB threshold values by ±5 points across all PROMs. This adjustment range was selected as a feasible approach, given the limited availability of reported CID and SCB values for each PROM specifically relevant to cartilage repair surgery in the existing literature. This approach aims to evaluate the sensitivity of the study's conclusions to potential variations in threshold values, thereby enhancing the reliability and applicability of the findings. Thereafter, for variables that were statistically significant in regression analyses, receiver operating characteristic (ROC) curve analyses were additionally performed to determine the optimal cut‐off value for the MOCART 2.0 score. Furthermore, to compare groups classified based on the cut‐off values identified in this study, Student's *t*‐test was used for continuous variables under the assumption of normal distribution according to the central limit theorem [14], while Pearson's chi‐squared test or Fisher's exact test was applied for categorical variables. The reliability of the radiological measurements (inter‐rater reliability) for continuous variables was assessed using intraclass correlation coefficients (ICCs) with a two‐way random‐effects model to determine absolute agreement between observers. A significance level of *p* < 0.05 was used.

## RESULTS

**Figure 1:**
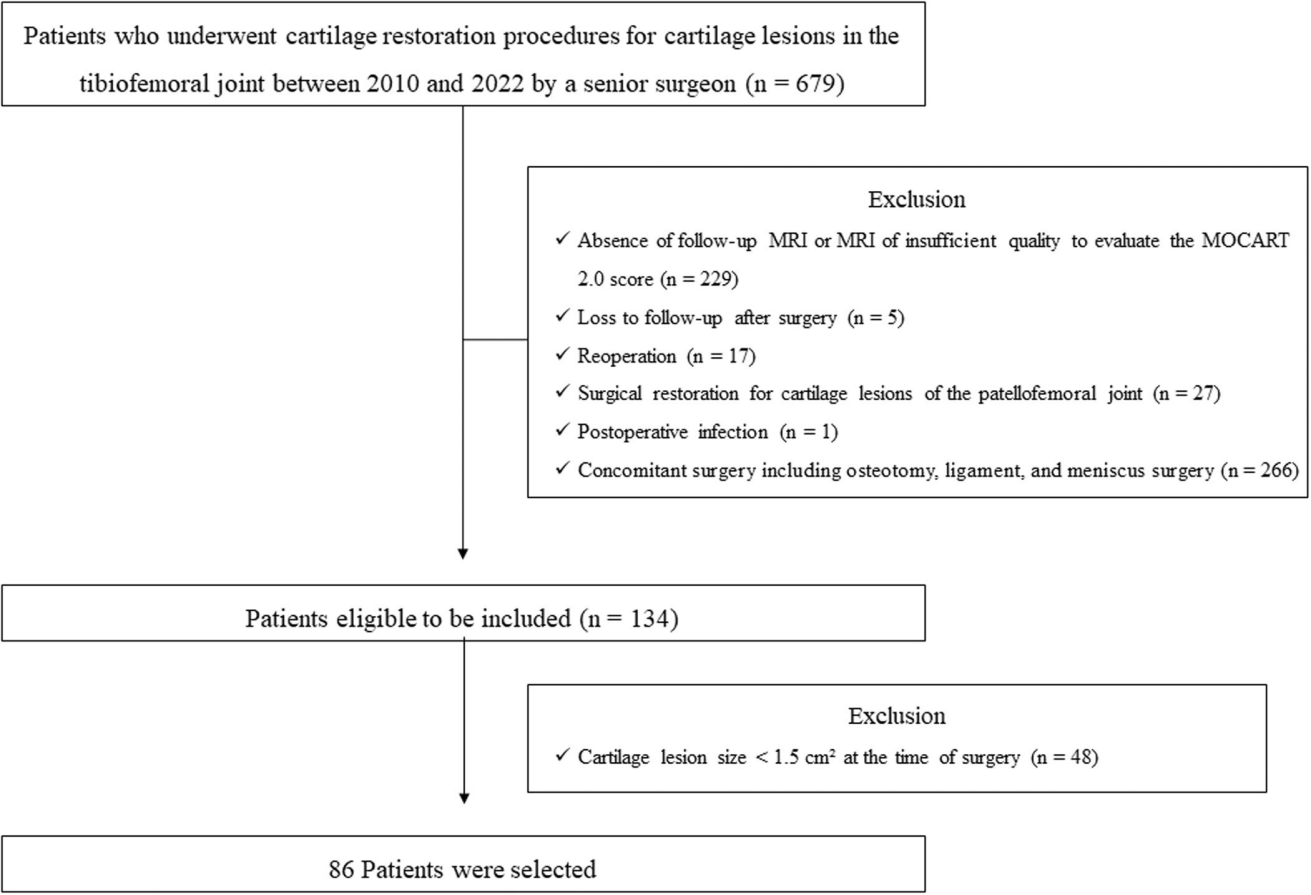
Patient flowchart. MOCART, Magnetic Resonance Observation of Cartilage Repair Tissue; MRI, magnetic resonance imaging.

A total of 86 patients were included in the study (Figure 1). The mean age of the patients was 52 ± 14.3 years, with 16 males and 70 females, and the mean follow‐up duration was 3 ± 1.9 years (range, 1–12 years) (Table S1). None of the included patients had bipolar cartilage lesions. The lesions were located in the medial compartment of the knee in 74 patients (86%) and in the lateral compartment in 12 patients (14%), and the mean size of the cartilage lesions at the time of surgery was 3.1 ± 1.5 cm^2^. The frequency of the different types of cartilage repair performed was as follows: MSP in 66 patients (77%), enhanced MSP in 5 patients (6%), ACI in 13 patients (15%) and MSC implantation in 2 patients (2%). The mean MOCART 2.0 score, evaluated using MRIs performed at 1 year post‐operatively, was 58 ± 14.3. Details of the overall cohort are provided in Table S1. The 95% confidence interval of the ICCs for the inter‐rater reliability in evaluating the MOCART 2.0 score was 0.861.

### Correlation and regression analyses

The correlation between the MOCART 2.0 score and PROMs at 1 year post‐operatively was analyzed. A statistically significant positive correlation was observed in most cases (*p* = 0.016 for the IKDC subjective form, *p* = 0.005 for the Lysholm score, *p* = 0.004 for KOOS symptoms, *p* = 0.006 for KOOS pain, *p* = 0.001 for KOOS activities of daily living and *p* = 0.023 for KOOS quality of life) (Table 2).

**Table 2:** Results of linear correlation analysis between MOCART 2.0 score and clinical scores 1 year after surgery.

Variables^a^	*r* b	*p*
IKDC subjective score	0.259	0.016
Lysholm score	0.301	0.005
KOOS symptoms	0.311	0.004
KOOS pain	0.295	0.006
KOOS activities of daily living	0.359	0.001
KOOS sports and recreation	0.173	0.111
KOOS quality of life	0.245	0.023

Abbreviations: IKDC, International Knee Documentation Committee; KOOS, Knee injury and Osteoarthritis Outcome Score; MOCART, Magnetic Resonance Observation of Cartilage Repair Tissue.

^a^ Clinical scores 1 year after surgery.

^b^ The correlation coefficient.

Logistic regression analysis was performed to determine whether the MOCART 2.0 scores were related to the achievement of clinically significant improvement following surgical repair for cartilage lesions in the tibiofemoral joint. The results showed that MOCART 2.0 scores were significantly associated with clinical improvements beyond the CID and SCB values for the Lysholm score, as well as with improvements beyond the SCB values for KOOS symptoms (*p* = 0.001 for the Lysholm score with respect to the CID value, *p* = 0.032 for the Lysholm score with respect to the SCB value, and *p* = 0.046 for KOOS symptoms with respect to the SCB value) (Table 3). These findings were consistently observed when analyzed using regression models with CID and SCB threshold values for each PROM adjusted by subtracting 5 points (*p* = 0.023 and 0.001 for the IKDC subjective score and KOOS symptoms for the CID value, respectively; *p* = 0.024 and 0.018 for the Lysholm score and KOOS symptoms for the SCB value) (Table S2). Likewise, comparable trends were noted when evaluating models with threshold values adjusted by adding 5 points (*p* = 0.007 and 0.046 for the Lysholm score and KOOS symptoms for the CID value, respectively; *p* = 0.025 for KOOS symptoms for the SCB value) (Table S3).

**Table 3:** Logistic regression analysis on the association between MOCART 2.0 score and classification based on clinical improvement beyond respective CID and SCB values 1 year post‐operatively.

Variables^a^	Beta coefficient	Odds ratio (95% CI)	*p*
Clinical improvement beyond respective CID value			
IKDC subjective score	−0.022	0.978 (0.954‒1.003)	0.083
Lysholm score	−0.053	0.949 (0.92‒0.978)	0.001
KOOS symptoms	−0.02	0.98 (0.956‒1.005)	0.116
KOOS pain	−0.012	0.988 (0.964‒1.013)	0.340
KOOS activities of daily living	−0.01	0.99 (0.967‒1.014)	0.431
KOOS sports and recreation	−0.014	0.986 (0.959‒1.014)	0.331
KOOS quality of life	−0.023	0.977 (0.946‒1.009)	0.161
Clinical improvement beyond respective SCB value			
IKDC subjective score	−0.047	0.954 (0.901‒1.011)	0.112
Lysholm score	−0.033	0.968 (0.939‒0.997)	0.032
KOOS symptoms	−0.028	0.972 (0.946‒0.999)	0.046
KOOS pain	−0.007	0.993 (0.968‒1.018)	0.576
KOOS activities of daily living	−0.022	0.978 (0.949‒1.009)	0.161
KOOS sports and recreation	−0.014	0.986 (0.959‒1.014)	0.331
KOOS quality of life	−0.015	0.985 (0.932‒1.041)	0.598

Abbreviations: CI, confidence interval; CID, clinically important difference; IKDC, International Knee Documentation Committee; KOOS, Knee injury and Osteoarthritis Outcome Score; MOCART, Magnetic Resonance Observation of Cartilage Repair Tissue; SCB, substantial clinical benefit.

^a^ Classification based on whether there was improvement beyond the respective CID and SCB values at 1 year post‐operatively compared to preoperative status.

### ROC analyses

**Figure 2:**
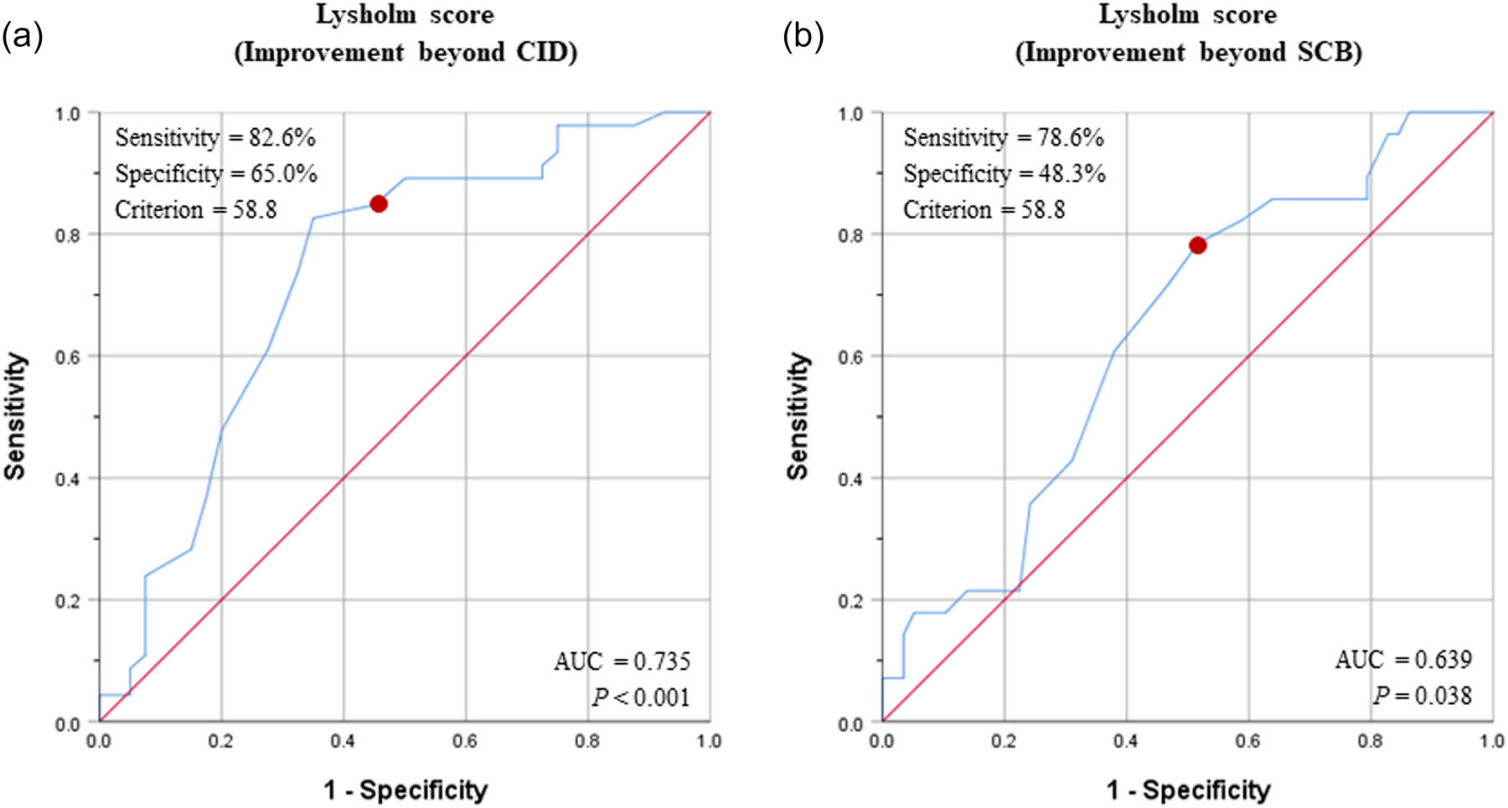
ROC curve analysis for the cut‐off value of the MOCART 2.0 score predicting clinical improvement beyond (a) the CID and (b) the SCB in the Lysholm score. AUC, area under the curve; CID, clinically important difference; MOCART, Magnetic Resonance Observation of Cartilage Repair Tissue; ROC, receiver operating characteristic; SCB, substantial clinical benefit.

Subsequently, for the variables that were statistically significant in the preceding regression analyses, ROC curves were plotted to determine the MOCART 2.0 score cut‐off value capable of distinguishing clinically important changes post‐operatively. The optimal cut‐off value was calculated to maximize both sensitivity and specificity. As a result, statistically significant cut‐off values were identified for the Lysholm score with respect to the CID and SCB values (*p* < 0.001 with respect to the CID value, *p* = 0.038 with respect to the SCB value), with the optimal threshold determined to be 58.8 in both cases. For the CID value, the accuracy was 73.5%, with a sensitivity of 82.6% and a specificity of 65.0%, while for the SCB value, the accuracy was 63.9%, with a sensitivity of 78.6% and a specificity of 48.3% (Figure 2). Furthermore, ROC analyses for cases that were statistically significant in additional regression analyses with adjusted CID and SCB threshold values revealed optimal thresholds for the MOCART 2.0 score ranging from 56.3 to 61.3 points (Figure S1).

### Between‐group comparisons

Finally, based on the cut‐off values for the MOCART 2.0 score identified in this study, patients were classified, and a comparative analysis was conducted on preoperative, intraoperative and post‐operative data. Although the analysis indicated a cut‐off range of 56.3–61.3 points, for practical application in clinical settings, a rounded cut‐off of 60 points was used, considering the structure of the MOCART 2.0 scoring system. The results showed that patients with MOCART 2.0 scores ≥60 demonstrated higher scores in the majority of PROMs at both the 1‐year post‐operative follow‐up and final follow‐up, as well as lower grades of osteoarthritis (Table 4).

**Table 4:** Comparative analysis between groups classified by cut‐off value for clinically relevant MOCART 2.0 score.

Variables^a^	MOCART 2.0 score ≥60 (*n* = 52)	MOCART 2.0 score <60 (*n* = 34)	*p*
Demographic data			
Age, years	52.2 ± 13.9	51.6 ± 15.1	0.852
Sex, male/female^b^	9/43	7/27	0.780
Body mass index, kg/m^2^	24.8 ± 4.0	24.8 ± 3.5	0.987
Affected side, right/left^b^	28/24	15/19	0.509
Follow‐up duration, years	2.7 ± 1.8	2.6 ± 2.1	0.73
Preoperative data			
IKDC subjective score	35.2 ± 13.2	38.3 ± 15.5	0.325
Lysholm score	42.9 ± 20.5	48.6 ± 22.3	0.231
KOOS symptoms	52.1 ± 15.2	56.9 ± 20.9	0.215
KOOS pain	52.6 ± 19.0	51.9 ± 21.1	0.867
KOOS activities of daily living	58.9 ± 18.9	57.8 ± 20.6	0.810
KOOS sports and recreation	24.4 ± 19.6	25.4 ± 24.1	0.830
KOOS quality of life	30.4 ± 19.5	32.4 ± 20.5	0.660
Kellgren–Lawrence grade, 0/1/2/3^b^	33/19/0/0	14/14/5/1	0.004
Hip–knee–ankle angle, °	1.9 ± 3.1	2.5 ± 3.7	0.373
Intra‐operative data			
Affected compartment			
Medial/lateral	45/7	29/5	>0.999
Cartilage lesion size, cm^2^	2.9 ± 1.5	3.4 ± 1.5	0.161
Type of cartilage repair surgery, MSP/enhanced MSP/ACI/MSC implantation^b^	41/2/8/1	25/3/5/1	0.771
Medial meniscus			
Functional/non‐functional/repair	30/0/22	19/0/15	>0.999
Lateral meniscus			
Functional/non‐functional/repair	51/0/1	33/0/1	>0.999
Post‐operative 1 year			
IKDC subjective score	51.9 ± 13.1	47.8 ± 16.8	0.206
Lysholm score	66.4 ± 18.2	53.2 ± 25.2	0.011
KOOS symptoms	69.4 ± 16.8	58.9 ± 17.4	0.007
KOOS pain	73.0 ± 15.2	63.6 ± 20.2	0.025
KOOS activities of daily living	76.2 ± 15.5	64.0 ± 19.8	0.002
KOOS sports and recreation	42.1 ± 25.0	34.1 ± 22.6	0.136
KOOS quality of life	41.3 ± 20.1	30.7 ± 18.5	0.016
Kellgren–Lawrence grade, 0/1/2/3^b^	19/25/8/0	6/14/11/3	0.017
At final follow‐up			
IKDC subjective score	54.4 ± 14.5	51.2 ± 17.7	0.365
Lysholm score	68.9 ± 18.6	55.6 ± 25.4	0.011
KOOS symptom	68.6 ± 19.0	61.6 ± 18.0	0.089
KOOS pain	73.1 ± 16.4	63.6 ± 21.9	0.034
KOOS activities of daily living	78.7 ± 16.8	67.7 ± 20.5	0.012
KOOS sports and recreation	44.2 ± 24.4	38.8 ± 27.2	0.339
KOOS quality of life	44.6 ± 20.1	36.9 ± 20.5	0.091
Kellgren–Lawrence grade, 0/1/2/3^b^	15/24/12/1	5/7/16/6	0.001

Abbreviations: ACI, autologous chondrocyte implantation; IKDC, International Knee Documentation Committee; KOOS, Knee injury and Osteoarthritis Outcome Score; MOCART, Magnetic Resonance Observation of Cartilage Repair Tissue; MSC, mesenchymal stem cell; MSP, marrow stimulation procedure.

^a^ The values are given as the mean and standard deviation, otherwise noted separately.

^b^ The values are given as number of patients.

## DISCUSSION

The principal finding of this study was that the MOCART 2.0 score, assessed one year after surgical repair of cartilage lesions in the tibiofemoral joint of the knee, was significantly associated with post‐operative clinical outcomes. Higher MOCART 2.0 scores corresponded with higher post‐operative PROMs. Notably, a MOCART 2.0 score of ≥60 was found to be predictive of more favourable clinical outcomes.

The MOCART 2.0 score is a well‐established semiquantitative assessment tool designed to objectively evaluate the morphological outcomes of surgical repair for cartilage lesions in the knee joint [24, 25]. It appropriately reflects recent advancements in cartilage repair techniques and MRI technology and is known for its high reproducibility [19, 24, 25]. However, there has been little research on the clinical relevance of this scoring system, and similarly, its interpretation in relation to clinical outcomes has not been investigated. Therefore, this study analyzed the association between the MOCART 2.0 scoring system and clinical outcomes following surgical repair of cartilage lesions in the tibiofemoral joint, as well as the relevant threshold values.

This study demonstrated that the MOCART 2.0 score, assessed one year after surgical repair for tibiofemoral joint cartilage lesions, has a positive correlation with clinical outcomes. This was observed in the IKDC subjective score, Lysholm score and in most subscales of the KOOS. Recently, some studies have analyzed the relationship between the MOCART 2.0 score and clinical outcomes, and, similar to the findings of this study, they reported a positive correlation between morphological assessment and clinical outcomes [4, 22]. However, their research focused on cartilage lesions in the patellofemoral joint, and thus may not be applicable to cartilage lesions in the tibiofemoral joint, which are influenced by different joint kinematics and exhibit differing clinical characteristics. Therefore, this study has the strength of being the first to evaluate the relationship between morphological assessment using the MOCART 2.0 score and clinical outcomes after cartilage repair for tibiofemoral joint lesions. Additionally, the use of various PROMs in this study enhances its clinical relevance. Based on prior research and the novel findings of this study [4, 22], it can be summarized that higher MOCART 2.0 scores in the knee joint are associated with more favourable clinical outcomes.

In addition to the correlation analysis, this study also investigated the cut‐off value of the MOCART 2.0 score that could predict clinically significant improvement following cartilage repair. The analysis revealed that a score ranging from 56.3 to 61.3, but more practically, ≥60 points when considering its scoring system [24], was associated with clinically significant improvement post‐operatively. Regrettably, these findings did not apply to all PROMs. Nevertheless, this analysis was primarily conducted to identify the threshold MOCART 2.0 score capable of distinguishing clinical improvement after surgery, making its relevance to specific PROM a secondary consideration. Importantly, this study evaluated the threshold using two distinct criteria—CID and SCB—which reflect clinically meaningful changes, not just minimal improvements, further supporting the clinical relevance of the identified cut‐off value [3, 17, 21, 23]. Moreover, additional comparisons between groups classified by the 60‐point threshold for the MOCART 2.0 score revealed significant differences in most post‐operative clinical outcomes, including the radiographic grade of osteoarthritis. Interestingly, differences in Kellgren–Lawrence grades were evident even at the preoperative stage, with patients in the lower MOCART 2.0 score group tending to exhibit more advanced degenerative changes in the knee. This observation suggests a potential association between preoperative Kellgren–Lawrence grades and MOCART 2.0 scores. However, it is important to note that, while this potential relationship warrants further investigation, the primary aim of this study was to evaluate the association between MOCART 2.0 scores and postoperative clinical outcomes. Therefore, any potential correlation between preoperative Kellgren–Lawrence grades and MOCART 2.0 scores should not detract from the study's primary findings. In light of the findings from the aforementioned analyses, it can be suggested that after surgical repair for tibiofemoral joint cartilage lesions, a MOCART 2.0 score of ≥60 is indicative of favourable clinical outcomes.

Despite its potential advantages, the clinical utility of the MOCART 2.0 score has remained limited. Although many researchers have used this scoring system to evaluate post‐operative outcomes, a standardized method for its clinical interpretation has not yet been established. Encouragingly, through this study, the clinical significance of morphological assessment following surgical repair for tibiofemoral joint cartilage lesions was validated, and a method for interpreting the MOCART 2.0 score in relation to clinical outcomes was identified. The findings of this study could serve as a clinical guideline for evaluating clinical outcomes after cartilage repair.

The limitations of this study are as follows. First, as this was a retrospective study, there was a potential risk of bias during the evaluation process. Second, the relatively small number of patients included in this study warrants consideration. During the early phase of the study period, follow‐up MRIs were not actively recommended to patients, and a considerable number of MRIs were performed at external institutions. Consequently, a substantial portion of patients had to be excluded. Moreover, patients who underwent concomitant surgical procedures were excluded to focus exclusively on the clinical outcomes of cartilage repair. While this exclusion was methodologically necessary, it significantly reduced the study population and may have introduced a potential risk of selection bias. Third, the study included patients who had undergone various types of cartilage repair. Notably, marrow stimulation procedures were performed in the majority of patients included in this study. While the primary objective was to analyze the relationship between the MOCART 2.0 score and clinical outcomes, the different types of cartilage procedures may have influenced both the measurement of the MOCART 2.0 score and the clinical outcomes. In addition, the potential influence of defect size on these outcomes was not specifically analyzed, as the study was not designed to compare subgroups according to lesion size. However, previous reports have indicated that the MOCART 2.0 score demonstrates high measurement reliability regardless of the cartilage repair technique used [25]. Fourth, the associations between each subscale that constitutes the MOCART 2.0 score and PROMs were not analyzed. However, considering the purposes of this study, such overly detailed classifications could risk diverting focus, and investigating this matter should appropriately be carried out through follow‐up studies.

## CONCLUSION

The MOCART 2.0 score, assessed one year after surgical repair for cartilage lesions in the tibiofemoral joint, positively correlates with PROMs, with scores of ≥60 expected to be associated with favourable clinical outcomes.

## AUTHOR CONTRIBUTIONS

The project was coordinated by Sung‐Hwan Kim, while Hyun‐Soo Moon conceptualized the study. Hyun‐Soo Moon drafted the manuscript with contributions from Min Jung, Kwangho Chung and Se‐Han Jung. Data acquisition and analysis were conducted by Jin‐Gyu Kim and Min‐Cheol Park. Sungjun Kim and Chong‐Hyuk Choi provided supervision and guidance throughout the research process. All authors jointly contributed to the study design and data interpretation. Hyun‐Soo Moon critically revised the final draft for important intellectual content, and Sung‐Hwan Kim approved the final version for submission. All authors are aware of and agree to be accountable for all aspects of the work, ensuring that any questions related to the accuracy or integrity of any part of the work are appropriately investigated and resolved.

## CONFLICT OF INTEREST STATEMENT

The authors declare no conflicts of interest.

## ETHICS STATEMENT

This study was ethically approved by the Institutional Review Board from Severance Hospital (ID Number: 4‐2024‐0996).

## Supporting information

Supporting information.

Supplementary Table 1 Demographic and clinical data for the overall cohort. Supplementary Table 2 Logistic regression analysis on the association between MOCART 2.0 score and classification based on clinical improvement beyond thresholds adjusted by ‐5 points from respective CID and SCB values 1 year postoperatively. Supplementary Table 3 Logistic regression analysis on the association between MOCART 2.0 score and classification based on clinical improvement beyond thresholds adjusted by +5 points from respective CID and SCB values 1 year postoperatively.

## Data Availability

The data sets analyzed during the current study are available on reasonable request.
