# Supplementary material for: MOCART 2.0 score of 60 or greater measured at 1 year post‐operatively predicts favourable clinical outcomes after surgical repair of tibiofemoral cartilage lesions
Source: Knee Surg Sports Traumatol Arthrosc. 2025 Oct 15;34(9):3247–56. doi: 10.1002/ksa.70086 (PMC13502430; doi:10.1002/ksa.70086)
Supplement: Supplementary file 1 [file KSA-34-3247-s001.pdf]

PE Queries

Article Number: **Editor Name:**

In Case of Keying or conversion

1.
2.
3.
4.
5.
6.

Front matter Queries

1.
2.
3.
4.
5.
6.
7.
8.

References Queries

1.
2.
3.
4.
5.
6.
7.
8.
9.
10.

Original	&	Modified
1.	TO	
2.	TO	
3.	TO	
4.	TO	

References Queries

	ORIGINAL		MODIFIED
5.		TO	
6.		TO	
7.		TO	
8.		TO	
9.		TO	
10.		TO	
11.		TO	
12.		TO	
13.		TO	
14.		TO	

15. TO

16. TO

17. TO

18. TO

OTHER QUERIES
