# Supplementary material for: MOCART 2.0 score of 60 or greater measured at 1 year post‐operatively predicts favourable clinical outcomes after surgical repair of tibiofemoral cartilage lesions
Source: Knee Surg Sports Traumatol Arthrosc. 2025 Oct 15;34(9):3247–56. doi: 10.1002/ksa.70086 (PMC13502430; doi:10.1002/ksa.70086)
Supplement: Supplementary file 2 [file KSA-34-3247-s002.docx]

**Supplementary Table 1** Demographic and clinical data for the overall cohort

| Variables |  |
| --- | --- |
| Demographic Data |  |
| Age, years | 51.9 ± 14.3 |
| Sex, male/female | 16/70 |
| Body mass index, kg/m^2^ | 24.8 ± 3.8 |
| Affected side, right/ left | 43/43 |
| Follow-up duration, years | 2.7 ± 1.9 |
| Preoperative Data |  |
| IKDC subjective score | 36.4 ± 14.2 |
| Lysholm score | 45.2 ± 21.3 |
| KOOS Symptoms  KOOS Pain | 54.0 ± 17.7 |
|  | 52.3 ± 19.7 |
| KOOS Activities of Daily Living | 58.5 ± 19.5 |
| KOOS Sports and Recreation | 24.8 ± 21.4 |
| KOOS Quality of Life | 1.2 ± 19.8 |
| Kellgren-Lawrence grade, 0/1/2/3 | 47/33/5/1 |
| Hip-Knee-Ankle angle, ° | 2.1 ± 3.3 |
| Intra-operative Data |  |
| Affected compartment |  |
| Medial/ Lateral | 74/12 |
| Cartilage lesion size, cm^2^ | 3.1 ± 1.5 |
| Type of cartilage repair surgery |  |
| MSP/ Enhanced MSP/ ACI/ MSC implantation | 66/5/13/2 |
| Medial meniscus |  |
| Functional/ Non-functional/ Repair | 49/0/37 |
| Lateral meniscus |  |
| Functional/ Non-functional/ Repair | 84/0/2 |
| Postoperative 1-year |  |
| IKDC subjective score | 50.3 ± 14.7 |
| Lysholm score | 61.2 ± 22.0 |
| KOOS Symptoms | 65.3 ± 17.7 |
| KOOS Pain | 69.3 ± 17.8 |
| KOOS Activities of Daily Living | 71.4 ± 18.3 |
| KOOS Sports and Recreation | 39.0 ± 24.3 |
| KOOS Quality of Life | 37.1 ± 20.0 |
| Kellgren-Lawrence grade, 0/1/2/3 | 25/39/19/3 |
| MOCART 2.0 score | 58.4 ± 18.0 |
| At final follow-up |  |
| IKDC subjective score | 53.1 ± 15.8 |
| Lysholm score | 63.7 ± 22.4 |
| KOOS Symptoms | 65.8 ± 18.8 |
| KOOS Pain | 69.3 ± 19.2 |
| KOOS Activities of Daily Living | 74.4 ± 19.0 |
| KOOS Sports and Recreation | 42.1 ± 25.5 |
| KOOS Quality of Life | 41.6 ± 20.5 |
| Kellgren-Lawrence grade, 0/1/2/3 | 20/31/28/7 |

IKDC, International Knee Documentation Committee; KOOS, Knee injury and Osteoarthritis Outcome Score; MSP, Marrow stimulation procedure; ACI, autologous chondrocyte implantation; MSC, mesenchymal stem cell; MOCART, Magnetic Resonance Observation of Cartilage Repair Tissue.

**Supplementary Table 2** Logistic regression analysis on the association between MOCART 2.0 score and classification based on clinical improvement beyond thresholds adjusted by -5 points from respective CID and SCB values 1 year postoperatively

| Variables*^a^* | Beta coefficient | Odds ratio (95% CI) | *P*-value |
| --- | --- | --- | --- |
| Clinical improvement beyond respective CID value - 5 points |  |  |  |
| IKDC subjective score | -0.032 | 0.969 (0.942 ‒ 0.996) | 0.023 |
| Lysholm score | -0.021 | 0.979 (0.955 ‒ 1.004) | 0.094 |
| KOOS Symptoms | -0.049 | 0.952 (0.925 ‒ 0.98) | 0.001 |
| KOOS Pain | -0.004 | 0.996 (0.971 ‒ 1.022) | 0.758 |
| KOOS Activities of Daily Living | -0.02 | 0.98 (0.956 ‒ 1.005) | 0.120 |
| KOOS Sports and Recreation | -0.024 | 0.976 (0.949 ‒ 1.004) | 0.095 |
| KOOS Quality of Life | -0.013 | 0.987 (0.961 ‒ 1.013) | 0.317 |
| Clinical improvement beyond respective SCB value - 5 points |  |  |  |
| IKDC subjective score | -0.04 | 0.961 (0.921 ‒ 1.002) | 0.064 |
| Lysholm score | -0.031 | 0.969 (0.943 ‒ 0.996) | 0.024 |
| KOOS Symptoms | -0.031 | 0.969 (0.944 ‒ 0.995) | 0.018 |
| KOOS Pain | -0.011 | 0.989 (0.946 ‒ 1.014) | 0.376 |
| KOOS Activities of Daily Living | -0.012 | 0.988 (0.963 ‒ 1.014) | 0.374 |
| KOOS Sports and Recreation | -0.024 | 0.976 (0.949 ‒ 1.004) | 0.095 |
| KOOS Quality of Life | -0.024 | 0.976 (0.928 ‒ 1.026) | 0.339 |

MOCART, Magnetic Resonance Observation of Cartilage Repair Tissue; CID, clinically important difference; SCB, substantial clinical benefit; IKDC, International Knee Documentation Committee; KOOS, Knee injury and Osteoarthritis Outcome Score.

*^a^*Classification based on whether there was improvement beyond the threshold, adjusted by -5 points from respective CID and SCB values, at 1 year postoperatively compared to the preoperative status.

**Supplementary Table 3** Logistic regression analysis on the association between MOCART 2.0 score and classification based on clinical improvement beyond thresholds adjusted by +5 points from respective CID and SCB values 1 year postoperatively

| Variables*^a^* | Beta coefficient | Odds ratio (95% CI) | *P*-value |
| --- | --- | --- | --- |
| Clinical improvement beyond respective CID value + 5 points |  |  |  |
| IKDC subjective score | -0.022 | 0.978 (0.954 ‒ 1.003) | 0.085 |
| Lysholm score | -0.038 | 0.963 (0.937 ‒ 0.99) | 0.007 |
| KOOS Symptoms | -0.028 | 0.972 (0.946 ‒ 0.999) | 0.046 |
| KOOS Pain | -0.012 | 0.988 (0.965 ‒ 1.012) | 0.342 |
| KOOS Activities of Daily Living | -0.011 | 0.989 (0.965 ‒ 1.013) | 0.373 |
| KOOS Sports and Recreation | -0.025 | 0.975 (0.945 ‒ 1.007) | 0.124 |
| KOOS Quality of Life | -0.023 | 0.977 (0.946 ‒ 1.009) | 0.161 |
| Clinical improvement beyond respective SCB value + 5 points |  |  |  |
| IKDC subjective score | -0.063 | 0.939 (0.867 ‒ 1.018) | 0.126 |
| Lysholm score | -0.023 | 0.977 (0.944 ‒ 1.011) | 0.190 |
| KOOS Symptoms | -0.033 | 0.967 (0.94 ‒ 0.996) | 0.025 |
| KOOS Pain | -0.008 | 0.992 (0.964 ‒ 1.021) | 0.588 |
| KOOS Activities of Daily Living | -0.018 | 0.982 (0.948 ‒ 1.017) | 0.312 |
| KOOS Sports and Recreation | -0.025 | 0.975 (0.945 ‒ 1.007) | 0.124 |
| KOOS Quality of Life | -0.015 | 0.985 (0.932 ‒ 1.041) | 0.598 |

MOCART, Magnetic Resonance Observation of Cartilage Repair Tissue; CID, clinically important difference; SCB, substantial clinical benefit; IKDC, International Knee Documentation Committee; KOOS, Knee injury and Osteoarthritis Outcome Score.

*^a^*Classification based on whether there was improvement beyond the threshold, adjusted by +5 points from respective CID and SCB values, at 1 year postoperatively compared to the preoperative status.
